# Machine Learning for Biomedical Literature Triage

**DOI:** 10.1371/journal.pone.0115892

**Published:** 2014-12-31

**Authors:** Hayda Almeida, Marie-Jean Meurs, Leila Kosseim, Greg Butler, Adrian Tsang

**Affiliations:** 1 Department of Computer Science and Software Engineering, Concordia University, Montreal, QC, Canada; 2 Centre for Structural and Functional Genomics, Concordia University, Montreal, QC, Canada; University of Westminster, United Kingdom

## Abstract

This paper presents a machine learning system for supporting the first task of the biological literature manual curation process, called triage. We compare the performance of various classification models, by experimenting with dataset sampling factors and a set of features, as well as three different machine learning algorithms (Naive Bayes, Support Vector Machine and Logistic Model Trees). The results show that the most fitting model to handle the imbalanced datasets of the triage classification task is obtained by using domain relevant features, an under-sampling technique, and the Logistic Model Trees algorithm.

## Introduction

Databases allows storing data in a consistent way, facilitating easy retrieval and enabling both complex searches and computation on data. In the biomedical field, databases are also used as vital resources for scientists searching literature. Over the past few years, researchers and users have noted a significant expansion of such literature databases [1]. For example, the free on-line database PubMed [2] currently holds over 22 million documents, and a simple keyword search can retrieve more than hundreds of thousands of documents. The analysis of the vast biomedical data currently available is a challenge addressed by studies such as [5] [6], as well as the use of this data to identify relevant information for biomedical research [7] [8]. Biocurators who seek relevant information to populate biomedical databases usually go through a time-consuming and error-prone process, named triage [3]. The triage process requires querying the document collection for keywords, and filtering among a long list of results for only the documents that seem to be potential candidates for full curation. This first triage step creates a severe bottleneck in the manual curation workflow [3] [22], and therefore could greatly benefit from automatic support.

**Figure 1:**
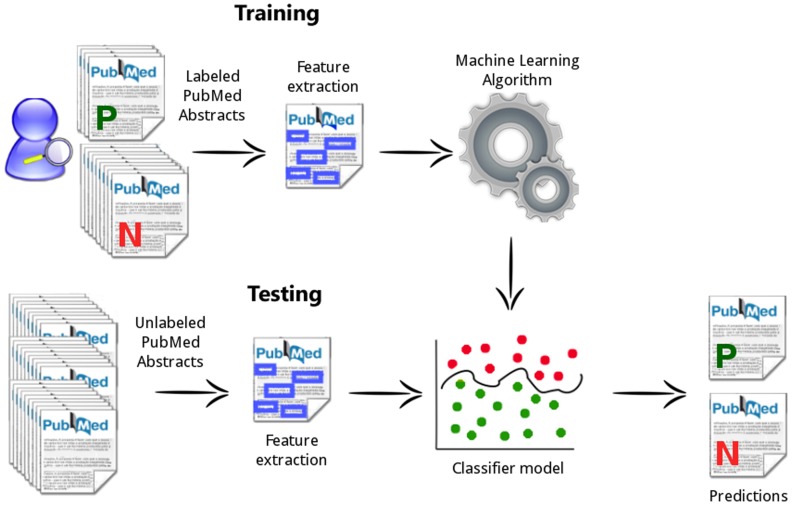
System Workflow. mycoSORT Training and Testing processes.

In this paper, we present a supervised machine learning approach to perform text classification of PubMed abstracts, with the goal of supporting the triage of documents. As shown in Fig. 1, in the training phase, the system learns from correctly labeled samples of abstracts, and makes a classification decision on a new (tested) PubMed abstract based on the analysis of specific features of the data, such as relevant pieces of text that can represent biological entities, frequency of keywords or alpha-numerical identifiers.

By nature, the classification scenario of the triage task is highly imbalanced, since the task consists in retrieving few very specific publications among the often huge volume of available articles. In our context, curators are looking for reference articles related to characterized lignocellulose-active proteins of fungal origin that will populate the mycoCLAP database [4]. The presence of relevant documents is limited to an average representation of only 10% of the total set of retrieved documents. In imbalanced scenarios, classification algorithms are naturally biased by the distribution of documents, and therefore tend to favor the majority class and overlook the minority class instances.

In this paper, we discuss the use of 108 different classification models, defined by experimenting with feature settings, classifiers and class distributions achieved through data sampling. Our goal is to determine the most fitting model, capable of dealing with the imbalanced data issue representative of a real life task and achieving satisfying results.

Machine learning from imbalanced data is a common problem of many real world applications beyond genomics text classification [9], such as fraud detection [10] [11], medical diagnosis [12] [13] and speech recognition [14]. The imbalance issue can interfere directly on the classifier performance, which is biased by the majority class. Because the majority class is more heavily represented in the dataset than the minority class, it tends to have more influence under uncertainty cases, since the class distribution can influence learning criteria. In addition, according to [15], a classifier presents a lower error rate when classifying an instance belonging to the majority class, since it will have learned more information from the examples of the majority class, compared to the information learned in fewer examples from the minority class. As classification algorithms tend to maximize the overall accuracy, the misclassification errors are equally considered. This implies that a majority instance misclassified as a minority one has the same error cost than a minority instance misclassified as a majority one. Because the minority class is so little represented in the dataset, even if a classifier assigns a majority class label to all minority instances, the overall accuracy would still be fairly acceptable. However, this high accuracy does not mean that minority class instances, the most relevant ones, are being correctly classified by a given model. Various approaches have attempted to overcome the imbalanced data issue. Two widely known approaches are, at the algorithm level, the use of cost-sensitive classifiers [16], and at the data level, dataset sampling methods [17]. Cost sensitive classifiers minimize classification errors on the minority class by biasing the classifier towards making mistakes on the majority class instead. The algorithm learns that an error made on the minority class is more costly than an error made on the majority class. At the data level, the Synthetic Minority Over-sampling Technique (SMOTE) [17] suggests a combination of under-sampling (i.e. reducing the majority class) and over-sampling (i.e. generating synthetic examples of the minority class), in an attempt to balance the minority class distribution.

A comparative study [18] between cost-sensitive and sampling methods was not conclusive about the best approach to handle imbalanced data. Still, the authors indicate that the class imbalance characteristic is an important factor to be taken into account, because it may affect the sampling factors that are exploited. In this previously described work, the authors adopted Decision Trees (C4.5) [21] and Naïve Bayes as classification algorithms.

Several studies have evaluated the performance of Support Vector Machine (SVM) [19] to handle the imbalance issue, and described it as a sensitive algorithm to skewed corpora. Akbani et al. [20] described a technique that combines SVM and over-sampling, called SMOTE with Different Costs (SDC). The results of the SDC system showed a better performance compared to a standard SVM implementation or compared to an under-sampling method to equalize the classes distribution. Yet, the authors clarify that the SDC algorithm makes the assumption that the minority instances are similar in content and found close to each other on the dimensional space, conditions that should not be considered by all means as typical.

Going beyond the standard SVM model, Tang et al. [23] demonstrated a generally better performance of Granular SVMs (GSVM), considering the application of an under-sampling method, compared to other variations of the SVM model. However, the GSVM was formerly described as a method likely to overfit [24].

The work of Mountassir et al. [25] evaluated random under-sampling with variations of under-sampling methods on imbalanced corpora. They compared the performance of classification models using standard implementations of SVM, Naïve Bayes and k-Nearest Neighbor (k-NN). Their conclusions showed that SVM was the most sensitive classifier to imbalanced corpora. In addition, all variations of under-sampling methods performed similarly on the most imbalanced datasets used in the experiments (in which the minority class was represented by 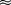 8% of the total number of instances).

In a text classification challenge, Charton et al. [26] achieved a high performance when dealing with severely imbalanced data. Their system was able to handle the classification of minority classes (that were represented by 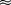 8% and even 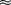 0.6% of the total number of instances of the dataset) on a 4-class corpus, and outperformed all the other systems participating in the same task. The solution presented by the authors was a model formed by a combination of feature types, and the use of the Logistic Model Trees (LMT) [27] classifier. The system also showed better performance when evaluated against other classifiers, such as Naïve Bayes, Decision Trees, as well as SVM.

In this paper, we present a similar approach to [26] to tackle the problem of triage classification. In general, the previously described works made use of a readily available corpus, usually suitable for general tasks. In our work, we build and adopt a specific corpus, specifically designed for the triage task, and we discuss an approach focusing on dataset sampling and feature settings.

This paper is structured as follows: Section **Materials and Methods** is formed by subsections **Corpus** and **Methodology**. The subsection **Corpus** describes the characteristics of the dataset used in our experiments. The subsection **Methodology** introduces the approaches used to build the classification models, describing the algorithms, the data representation and evaluation metrics used in the experiments. In Section **Results**, we present and discuss our findings after experimenting with 3 classifiers, 5 feature types and 9 sampling factors. Finally, Section **Discussion** presents our analysis, and Section **Conclusion** summarizes our work and future research avenues.

## Materials and Methods

### Corpus

#### Corpus Creation

The dataset employed in our experiments is composed of PubMed abstracts retrieved by biocurators using specific queries and time range. Queries were built with the *name of an enzyme (family) of interest*, the logical conjunction *AND*, and the generic string fung* to match fungal-related terms. All abstracts were published before December 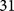, 2013. For instance, taking this period into account, the query {beta-glucosidase AND fung*} returns a list of 1296 related abstracts. The retrieved list of results was preprocessed with the mycoMINE text mining system [28], which added bio-entity annotations to relevant units of text.

All documents were then correctly labeled by biocurators as belonging to one of two classes, which indicate if the document will be selected or not for the full curation process. Relevant documents are considered to belong to the *positive* class and will be retained for full curation; while non-relevant documents are considered to belong to the *negative* class, and will be rejected by biocurators.

After the manual labeling effort, biocurators were able to identify paper abstracts related to a total of 28 enzyme families, which resulted in 749 positive documents. The equivalent number of rejected documents adds up to 6,834 negative instances.

Table 1 gathers some statistics on this corpus, which we call *mycoSet*. As shown in Table 1, the total number of instances is 7,583, and *mycoSet* is highly imbalanced. The majority class, which has the negative label, represents 90.12% of the total number of instances in the corpus, while the minority class, which has the positive label, is represented by only 9.88% of the instances.

**Table 1:** *mycoSet* Corpus Statistics.

Attribute	Quantity
Total # of instances	7,583 (100%)
Total # of abstracts with text content	6,898 (90.96%)
Negative instances	6,834 (90.12%)
Positive instances	749 (9.88%)
# of words in paper abstracts	43,598
# of words in paper titles	12,388
# of annotations in paper abstracts	50,866
# of annotations in paper titles	8,172
# of EC numbers	12,272

Statistics of the *mycoSet* corpus.

#### Training and Test Corpora

In order to build our classifiers, training and test corpora have been created from the *mycoSet* dataset. The training corpus is a fraction of the dataset used by the classification algorithms to learn a model that is able to distinguish instances by their class. The test corpus is a distinct fraction of the dataset that contains instance examples used to evaluate this model.

The test corpus was randomly created as 20.5% of the *mycoSet* dataset instances. We aim to evaluate our models on a corpus that represents a realistic class distribution.

The test set should therefore maintain an imbalanced distribution. This strategy allows the classifier to fit and evaluate a classification model that will be capable of handling the triage task in practice. Thus, we generated a test corpus that contains the same class distribution as in *mycoSet*, with 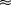 10% positive instances and 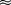 90% negative instances.

The training corpus was generated with the remaining instances of *mycoSet*. These remaining instances are not only highly imbalanced, but also numerous. As an effort to cope with both issues, a random sampling technique was used to create the training corpus. This process is further explained in Methodology.

### Methodology

#### Sampling

Sampling is a method used to deal with imbalanced data that generally involves low computational cost, since the data processing can be executed before the learning phase. Although sampling has not been shown to outperform other methods that deal with imbalanced data such as [16] and [29], it does not present limitations inherent to certain classifiers, as some other restrictive techniques do, such as cost-sensitive classification [18]. Under-sampling is a sampling technique that consists of reducing the number of instances of the majority class down to a certain percentage. According to [18], through under-sampling it is possible to reduce training time, or even make the training phase feasible if the task is dealing with very large training sets.

**Figure 2:**
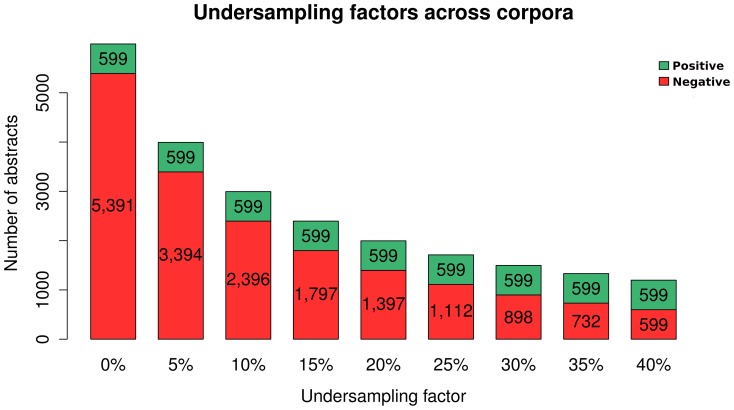
Corpora Under-sampling. Number of Instances and Balances across all Training Sets.

In this work, under-sampling was employed to build the training corpus, as a strategy to manage both the imbalanced and large size characteristics of the *mycoSet* corpus. In order to evaluate various training scenarios, we gradually and randomly eliminated a percentage of negative instances from *mycoSet*. Several training corpora were then generated through this progressive under-sampling approach. A variety of class distribution ratios provide an effective comparison between classifier performances at different bias degrees caused by the majority class. We started from a training corpus with a similar class distribution as in the *mycoSet* dataset. This allowed us to have a representative scenario of a real document triage. Then the number of negative instances was gradually reduced by a factor of 5%, until balance was achieved with similar distributions on both classes. This is shown in Fig. 2.

#### Features

In order to represent the dataset as a feature space, document instances in the dataset have to be expressed in fragments of useful information, and these are used as features to build the classification models. In our experiments, we extracted features from the paper abstract and title, respectively from the "AbstractText" and "ArticleTitle" text fields of PubMed, in addition to the Enzyme Commission (EC) numbers and the "RegistryNumber" text field.

#### Feature Extraction

Before performing feature extraction, the content gathered from each document was pre-processed. In this step, some ASCII special characters, such as punctuation, and extra blank spaces were removed. Likewise, unnecessary markup tags were eliminated from the content.

The dataset instances were then expressed by means of mycoMINE annotation content, their corresponding bio-entities and EC numbers. Bioentity annotations are grouped according to their span. We considered two different annotation spans: the first one takes into account an entire sentence; while the second span, which we call *entity*, is composed by a word or a group of words. Table 2 lists all the entities annotated by mycoMINE and their corresponding spans. Annotations belonging to the entity span group were pre-processed (as described above). Then, their content was kept as a feature, along with their corresponding entity. Sentence annotations were represented as a bag-of-words after being pre-processed. When representing annotation content as a bag-of-words, we discarded PubMed stop-words [30] and tokens with a length smaller than 3 characters. These very short tokens were eliminated because they contribute more to increase both the sparseness of the feature space and the learning time, than to improve the discriminative power of the classification models.

**Table 2:** *mycoSet* Bio-entities.

Entity	Span	Entity	Span
AccessionNumber	entity	Glycosylation	sentence
ActivityAssayConditions	sentence	Kinetics	sentence
Assay	entity	Laccase	entity
Buffer	entity	Lipase	entity
Characterization	entity	Peroxidase	entity
Enzyme	entity	pH	sentence
Expression	sentence	ProductAnalysis	sentence
Family	entity	Temperature	sentence
Fungus	entity	SpecificActivity	sentence
Gene	entity	Substrate	entity
GlycosideHydrolase	entity	SubstrateSpecificity	sentence

Bioentities and Spans in the *mycoSet* Corpus Annotated by the mycoMINE Text Mining System.

To give an example of annotation spans and feature representation, consider the following sample fragment from the *mycoSet* dataset, annotated with mycoMINE:

*<SubstrateSpecificity>The substrate specificity of three <Enzyme>ligninase </Enzyme> isozymes from the white-rot fungus <Fungus>Trametes versicolor</Fungus>has been investigated (…). </SubstrateSpecificity>(…) <RegistryNumber>EC 1.14.99.-</RegistryNumber>*

The following features are extracted from the above sentence:

• Bioentities of the entity span: *[ligninase, Enzyme]; [Trametes versicolor, fungus]*.
• Bioentities of the sentence span: *[substrate, substratespecificity]; [specificity, substratespecificity]; [three, substratespecificity]; [ligninase, substratespecificity]; [isozymes, substratespecificity]; [whiterot, substratespecificity]; [fungus, substratespecificity]; [trametes versicolor, substratespecificity]; [investigated, substratespecificity]*.
• EC number: *[11499]*.

These features were used to construct a feature vector, further explained in Subsection Feature Vector, that represents the data in both the training and the test datasets.

#### Feature Vector

Each document instance in both training and test datasets is represented as a vector of features. Let *I* be the number of document instances in a dataset, and *F* the number of extracted features. Each vector holds the number of feature occurrences across one document in a *F* 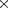 *I* matrix. For example, the document above would be roughly represented by the vector displayed in Table 3, in which the values represent the number of times a feature was seen in the text.

**Table 3:** *mycoSet* Feature Vector Representation.

ligninase	Trametes versicolor	synthetic	substrate	specificity	three	fungus	enzyme	…
2	2	1	1	1	1	2	1	…

Feature Occurrence Represented in the Feature Vector.

The larger the dataset size, the larger and sparser is the representation matrix. A sparse matrix reduces the accuracy of the classification models, while a large matrix can be costly in terms of computational processing during the training phase. Techniques to either reduce the dataset size, or the feature space through feature selection, can be valuable in these cases.

In this work, we explore a few standard feature selection methods in addition to sampling techniques. The features were selected according to their occurrence, as an effort to maintain a more compact feature space. Words occurring less than 2 times in the training corpus, or with less than 3 characters were not taken into account when generating feature vectors.

#### Classification Algorithms

For our experimental purposes, we considered three classification algorithms: Naïve Bayes (NB), Logistic Model Trees (LMT) and Support Vector Machine (SVM). A NB classifier is appropriate to provide a baseline evaluation of sampling and feature settings. LMT was previously described by [26] as an efficient classifier to handle tasks where datasets are imbalanced. An SVM is useful to provide a comparison between our model results and previous works that adopted this classifier to deal with imbalanced data. In the next sections we will briefly review these algorithms.

#### Naïve Bayes

A Naïve Bayes classifier is a probabilistic model based on Bayes' Rule, that assumes a strong conditional independence of features. This classifier builds a "Naïve" independence model, considering that in a feature vector 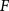, the features 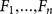 are conditionally independent from each other, given a class 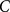. By this assumption, Naïve Bayes implies that the presence of one word (one feature) is not correlated with the presence or absence of another word in a document, considering a class label. Therefore, the probability of a document instance 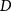 belonging to class 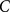, 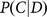, can be computed as:
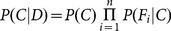
(1)where 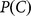 is the prior probability of a class 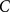, 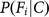 is the discriminative value of a feature 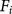 found within a document 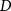 with regards to the class 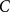, and 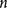 is the number of features. Naïve Bayes aims to identify the best 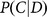, for all existing 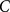. Hence, the classifier seeks to maximize a classification score for each document, as in:
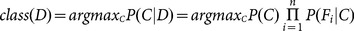
(2)where 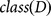 is the class value that maximizes 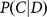. This value is defined after the class prior probability 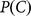 and each document feature value 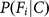 are computed.

#### Logistic Model Trees

Logistic Model Trees consist of a combination of Decision Tree and LogitBoost algorithms. A Logistic Model Tree is a classification tree, with logistic regression models on its nodes. At each node of the decision tree, the LogitBoost algorithm is used to train a data subset for a certain number of iterations. This number is defined through five fold cross validation. An error rate is computed at each iteration and the one presenting a lowest rate is selected to define a logistic regression model for the current node. A Decision Tree criterion is then applied to split the current data subset. A LogitBoost execution to be started at the child nodes will be initialized from the logistic regression model previously defined at the parent node. Tree splitting will be performed until there is still a relevant information gain.

In a Decision Tree model, leaves usually hold a class prediction as output. In a LMT model, leaves hold a logistic regression function for the current data subset at this node. A logistic function found in a LMT leaf forms a model that does not only represent the data within the current node. It is a model that has been continuously incremented, since it was built on top of a function first defined at the root node. The final model of a logistic model tree is defined as follows:
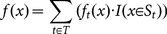
(3)where 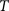 represents the set of all leaves (terminal nodes), 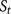 is the dataset split on the current leaf 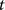, 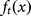 is the logistic regression function at the current node 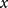. 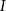 is the indicator function: the expression 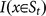 has a binary evaluation, returning 1 only when the instance 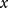 belongs to the current dataset split 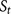.

#### Support Vector Machine

A Support Vector Machine (SVM) is a well known algorithm that converges to an optimal solution for linear and non-linear classifications. This classifier often outperforms many commonly used machine learning algorithms, even though it may not be an ideal choice to handle large datasets.

To separate data points on a dimensional space and tell their classes apart, SVM computes the "margin maximum classifier" [31]. A maximum margin is the largest radius around a classification boundary where no data points are placed. The closest data points encountered next to this margin are called support vectors. These vectors are considered as the hardest instances to be classified. Because of that, they are used as a "support" to draw a decision boundary and build a classification model. If a classification problem is identified as linearly separable, the data points are simply separated by a line in the space. When linear separation is not possible, SVM uses data transformation to separate the data point classes. The transformation computation is optimized to a linear decision with the use of a kernel function.

SVM classifies a new instance 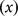 according to its distance from the support vectors 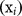, and also from the hyperplane, placed in the middle of a maximum margin. A weight vector is placed orthogonally to the hyperplane, and the class prediction 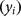 for a new instance represents its coefficient on the weight vector 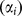. The decision function for SVM is computed as shown in the following equation:
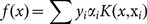
(4)where 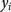 stands for the class prediction (+1 or −1 in a binary classification), 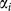 represents the weight vectors, 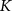 is the kernel function, 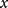 is the instance to be classified, and 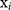 represents the support vectors.

#### Evaluation Metrics

Performance of classification algorithms can be displayed by a confusion matrix. As shown in Table 4, the confusion matrix of a classification output indicates the number of instances with regards to the predicted and the actual classes.

**Table 4:** *mycoSet* Confusion Matrix.

	Predicted Positive	Predicted Negative
**Positive class**	True Positive (TP)	False Negative (FN)
**Negative class**	False Positive (FP)	True Negative (TN)

Confusion Matrix of a Binary Classification.

To compare the performance of different classification models, we considered evaluation metrics that are not dependent on class distributions (the number of instances in each class), and therefore will not be biased by a imbalanced dataset. Experimental results of this work are presented by means of Precision, Recall, F-measure, F-2 and Matthews Correlation Coefficient (MCC). We briefly explain hereafter how each of these metrics is obtained from the confusion matrix scores, in a binary classification framework.

**Precision** evaluates the proportion of correct predictions among correct and incorrect predictions that the classifier makes for a certain class. This measure indicates if a classifier is capable of outputting more relevant than irrelevant results. Precision is calculated by the number of True Positives (TP, i.e. correctly classified documents) divided by the sum of True Positives and False Positives (TP and FP, i.e. all class predictions).
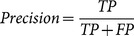
(5)


**Recall** represents the ratio of relevant predictions made by the classifier between all existing relevant instances that should have been predicted. This measure demonstrates the capability of a classifier to predict the universe of relevant instances. Recall is calculated by the number of TP (i.e. correctly classified documents) divided by TP plus False Negatives (FN) (i.e. all instances belonging to the same class).
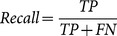
(6)


**F-measure** is the harmonic mean of Precision and Recall scores, obtained through the formula:
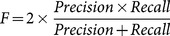
(7)


**F-** 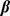 **score** is a generalization of the F-measure defined as follows:
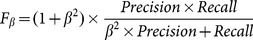
(8)where 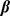 is the relative weight of Recall over Precision. Since in our experiments there is more interest in the model ability to identify the entire universe of relevant instances, Recall should be emphasized when calculating F-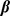 score. Thus, the 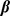 value should be greater than 1. In our experiments, we used 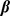  = 2, leading to the F-2 score.

**Matthews Correlation Coefficient** represents a coefficient of agreement between observed and predicted classifications. A correlation value equal to 1 stands for a total agreement (a perfect prediction), while a value equal to zero means total disagreement. MCC can be computed using the formula below:
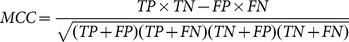
(9)


## Results

In this section, we present the experiments performed using several model configurations. We evaluate the performance of our proposed models when handling the imbalanced issue on the triage task using the *mycoSet* dataset.

### Experiments

#### Set of Features

The set of features used to build all classification models was derived from both PubMed abstracts, under the "AbstractText" field, and abstract titles, under the "ArticleTitle" field. After being pre-processed, as explained in the related Section above, the final set of features includes 5 types of features:

F1: Annotated bio-entities
F2: Annotated contents of entity spans
F3: Annotated contents of sentence spans (as a bag-of-words)
F4: Enzyme Commission (EC) numbers
F5: Bag-of-words representation of the entire fields (ArticleTitle and AbstractText)

#### Classifiers

The classifiers used in our experiments are built-in algorithm implementations available within the Weka framework [32]. The three classification algorithms previously described were used:

1. Naïve Bayes (NB)
2. Logistic Model Trees (LMT)
3. Support Vector Machine (LibSVM)

#### Under-sampling

The under-sampling technique was used to generate training corpora with different class distributions. A first dataset was created with a class distribution that is similar to the one present in a real triage classification scenario.

After generating this first training corpus, the number of negative instances in the corpus was gradually reduced to arrive at a more balanced distribution. Until the most balanced distribution was reached (i.e., when each class has the same amount of document instances), 9 under-sampling factors (USF) were employed.

1. Training set with 0% USF: 90% negative, 10% positive
2. Training set with 5% USF: 85% negative, 15% positive
3. Training set with 10% USF: 80% negative, 20% positive
4. Training set with 15% USF: 75% negative, 25% positive
5. Training set with 20% USF: 70% negative, 30% positive
6. Training set with 25% USF: 65% negative, 35% positive
7. Training set with 30% USF: 60% negative, 40% positive
8. Training set with 35% USF: 55% negative, 45% positive
9. Training set with 40% USF: 50% negative, 50% positive

This is shown in Fig. 2.

### Performance

We present here the results obtained after classifying the test set using the models built through our previously described approaches. Tables 5, 6, 7 and 8 present the Precision, Recall, F-Measure, F-2 and MCC results for the positive class, achieved with the three classifiers, using different feature settings.

**Table 5:** mycoSORT Results - Set of Features F1.

Under-sampling(USF)	Classifier	Precision	Recall	F-measure	MCC	F-2
Training set with USF 0%	Naive Bayes	0.286	0.227	0.253	0.182	0.240
Training set with USF 0%	LMT	0.492	0.207	0.291	0.274	0.230
Training set with USF 0%	LibSVM	0.714	0.033	0.064	0.140	0.04
Training set with USF 5%	Naive Bayes	0.294	0.280	0.287	0.210	0.280
Training set with USF 5%	LMT	0.461	0.233	0.310	0.278	0.260
Training set with USF 5%	LibSVM	0.645	0.133	0.221	0.264	0.160
Training set with USF 10%	Naive Bayes	0.269	0.307	0.287	0.202	0.300
Training set with USF 10%	LMT	0.376	0.213	0.272	0.226	0.230
Training set with USF 10%	LibSVM	0.47	0.207	0.287	0.264	0.230
Training set with USF 15%	Naive Bayes	0.301	0.347	0.322	0.241	0.340
Training set with USF 15%	LMT	0.352	0.413	0.380	0.307	0.400
Training set with USF 15%	LibSVM	0.387	0.287	0.330	0.271	0.300
Training set with USF 20%	Naive Bayes	0.263	0.340	0.297	0.209	0.320
Training set with USF 20%	LMT	0.348	0.480	0.403	0.331	0.450
Training set with USF 20%	LibSVM	0.353	0.353	0.353	0.281	0.350
Training set with USF 25%	Naive Bayes	0.243	0.353	0.288	0.197	0.320
Training set with USF 25%	LMT	0.286	0.547	0.375	0.301	0.460
Training set with USF 25%	LibSVM	0.282	0.413	0.335	0.251	0.380
Training set with USF 30%	Naive Bayes	0.277	0.440	0.340	0.257	0.390
Training set with USF 30%	LMT	0.291	0.627	0.397	0.334	0.510
Training set with USF 30%	LibSVM	0.258	0.48	0.336	0.252	0.410
Training set with USF 35%	Naive Bayes	0.242	0.440	0.312	0.223	0.380
Training set with USF 35%	LMT	0.233	0.620	0.338	0.266	0.470
Training set with USF 35%	LibSVM	0.210	0.633	0.316	0.241	0.450
Training set with USF 40%	Naive Bayes	0.254	0.467	0.329	0.243	0.400
Training set with USF 40%	LMT	0.269	0.660	0.382	0.321	0.510
Training set with USF 40%	LibSVM	0.196	0.667	0.303	0.229	0.450

Results of Positive Class on Feature Setting #1, Using Only Bio-entities as Features.

**Table 6:** mycoSORT Results - Set of Features F1+F4.

Under-sampling(USF)	Classifier	Precision	Recall	F-measure	MCC	F-2
Training set with USF 0%	Naive Bayes	0.285	0.380	0.326	0.242	0.360
Training set with USF 0%	LMT	0.516	0.107	0.177	0.202	0.130
Training set with USF 0%	LibSVM	1.000	0.020	0.039	0.134	0.020
Training set with USF 5%	Naive Bayes	0.273	0.373	0.315	0.230	0.350
Training set with USF 5%	LMT	0.426	0.173	0.246	0.224	0.200
Training set with USF 5%	LibSVM	0.833	0.033	0.064	0.155	0.040
Training set with USF 10%	Naive Bayes	0.268	0.427	0.329	0.243	0.380
Training set with USF 10%	LMT	0.412	0.233	0.298	0.255	0.260
Training set with USF 10%	LibSVM	0.688	0.073	0.133	0.203	0.090
Training set with USF 15%	Naive Bayes	0.268	0.427	0.329	0.243	0.380
Training set with USF 15%	LMT	0.398	0.300	0.342	0.284	0.320
Training set with USF 15%	LibSVM	0.604	0.193	0.293	0.306	0.220
Training set with USF 20%	Naive Bayes	0.275	0.440	0.338	0.255	0.390
Training set with USF 20%	LMT	0.322	0.393	0.354	0.276	0.380
Training set with USF 20%	LibSVM	0.471	0.327	0.386	0.338	0.350
Training set with USF 25%	Naive Bayes	0.258	0.507	0.342	0.260	0.420
Training set with USF 25%	LMT	0.321	0.520	0.397	0.324	0.460
Training set with USF 25%	LibSVM	0.364	0.420	0.390	0.318	0.410
Training set with USF 30%	Naive Bayes	0.237	0.540	0.329	0.248	0.430
Training set with USF 30%	LMT	0.328	0.500	0.396	0.322	0.450
Training set with USF 30%	LibSVM	0.323	0.473	0.384	0.308	0.430
Training set with USF 35%	Naive Bayes	0.227	0.513	0.315	0.229	0.410
Training set with USF 35%	LMT	0.267	0.587	0.367	0.295	0.470
Training set with USF 35%	LibSVM	0.251	0.573	0.349	0.274	0.460
Training set with USF 40%	Naive Bayes	0.244	0.520	0.332	0.250	0.420
Training set with USF 40%	LMT	0.267	0.707	0.388	0.334	0.530
Training set with USF 40%	LibSVM	0.217	0.613	0.321	0.245	0.450

Results of Positive Class on Feature Setting #2, Using Bio-entities and EC Numbers as Features.

**Table 7:** mycoSORT Results - Set of Features F5.

Under-sampling(USF)	Classifier	Precision	Recall	F-measure	MCC	F-2
Training set with USF 0%	Naive Bayes	0.307	0.720	0.430	0.382	0.570
Training set with USF 0%	LMT	0.656	0.420	0.512	0.485	0.450
Training set with USF 0%	LibSVM	0.833	0.033	0.064	0.155	0.040
Training set with USF 5%	Naive Bayes	0.310	0.733	0.436	0.390	0.580
Training set with USF 5%	LMT	0.600	0.500	0.545	0.503	0.520
Training set with USF 5%	LibSVM	0.703	0.173	0.278	0.319	0.200
Training set with USF 10%	Naive Bayes	0.307	0.760	0.438	0.396	0.590
Training set with USF 10%	LMT	0.574	0.567	0.570	0.523	0.570
Training set with USF 10%	LibSVM	0.704	0.333	0.452	0.449	0.370
Training set with USF 15%	Naive Bayes	0.309	0.793	0.445	0.41	0.600
Training set with USF 15%	LMT	0.458	0.693	0.552	0.504	0.630
Training set with USF 15%	LibSVM	0.596	0.413	0.488	0.451	0.440
Training set with USF 20%	Naive Bayes	0.314	0.793	0.450	0.415	0.610
Training set with USF 20%	LMT	0.422	0.653	0.513	0.460	0.590
Training set with USF 20%	LibSVM	0.545	0.527	0.536	0.485	0.530
Training set with USF 25%	Naive Bayes	0.312	0.780	0.446	0.408	0.600
Training set with USF 25%	LMT	0.399	0.673	0.501	0.449	0.590
Training set with USF 25%	LibSVM	0.481	0.580	0.526	0.470	0.560
Training set with USF 30%	Naive Bayes	0.288	0.767	0.418	0.377	0.580
Training set with USF 30%	LMT	0.388	0.727	0.506	0.461	0.620
Training set with USF 30%	LibSVM	0.460	0.687	0.551	0.503	0.630
Training set with USF 35%	Naive Bayes	0.302	0.780	0.435	0.397	0.590
Training set with USF 35%	LMT	0.359	0.807	0.497	0.465	0.650
Training set with USF 35%	LibSVM	0.369	0.800	0.505	0.472	0.650
Training set with USF 40%	Naive Bayes	0.303	0.773	0.435	0.396	0.590
Training set with USF 40%	LMT	0.344	0.840	0.488	0.463	0.650
Training set with USF 40%	LibSVM	0.338	0.840	0.482	0.456	0.650

Results of Positive Class on Feature Setting #3, Using Only Bag-of-Words as Features.

**Table 8:** mycoSORT Results - Set of Features F1+F2+F3+F4.

Under-sampling(USF)	Classifier	Precision	Recall	F-measure	MCC	F-2
Training set with USF 0%	Naive Bayes	0.355	0.727	0.477	0.431	0.600
Training set with USF 0%	LMT	0.685	0.420	0.521	0.498	0.460
Training set with USF 0%	LibSVM	0.867	0.087	0.158	0.257	0.110
Training set with USF 5%	Naive Bayes	0.365	0.740	0.489	0.446	0.610
Training set with USF 5%	LMT	0.585	0.480	0.527	0.484	0.500
Training set with USF 5%	LibSVM	0.729	0.287	0.411	0.424	0.330
Training set with USF 10%	Naive Bayes	0.349	0.787	0.484	0.448	0.630
Training set with USF 10%	LMT	0.552	0.600	0.575	0.526	0.590
Training set with USF 10%	LibSVM	0.670	0.420	0.516	0.491	0.450
Training set with USF 15%	Naive Bayes	0.342	0.787	0.477	0.441	0.620
Training set with USF 15%	LMT	0.478	0.647	0.550	0.498	0.600
Training set with USF 15%	LibSVM	0.607	0.473	0.532	0.491	0.490
Training set with USF 20%	Naive Bayes	0.342	0.793	0.478	0.443	0.630
Training set with USF 20%	LMT	0.425	0.64	0.511	0.456	0.580
Training set with USF 20%	LibSVM	0.521	0.587	0.552	0.500	0.570
Training set with USF 25%	Naive Bayes	0.322	0.787	0.457	0.421	0.610
Training set with USF 25%	LMT	0.389	0.747	0.511	0.469	0.630
Training set with USF 25%	LibSVM	0.474	0.667	0.554	0.504	0.620
Training set with USF 30%	Naive Bayes	0.336	0.773	0.469	0.430	0.610
Training set with USF 30%	LMT	0.398	0.780	0.527	0.490	0.650
Training set with USF 30%	LibSVM	0.459	0.673	0.546	0.496	0.620
Training set with USF 35%	Naive Bayes	0.304	0.800	0.440	0.406	0.600
Training set with USF 35%	LMT	0.343	0.760	0.473	0.433	0.610
Training set with USF 35%	LibSVM	0.357	0.793	0.493	0.458	0.640
Training set with USF 40%	Naive Bayes	0.295	0.780	0.428	0.389	0.590
Training set with USF 40%	LMT	0.361	0.847	0.506	0.481	0.670
Training set with USF 40%	LibSVM	0.331	0.793	0.468	0.433	0.620

Results of Positive Class on Feature Setting #4, Using Bio-entities, Content and EC Numbers as Features.

The results reported in Table 5 represent our feature setting #1, where the set of features is composed only by the 22 bio-entities, F1 as listed in Section Set of Features. In our feature setting #2 (see Table 6), the set of features is composed of the 22 bio-entities (F1) plus the EC numbers (F4) listed in the training set. The set of features in our feature setting #3 (see Table 7) is composed by the bag-of-words representation of the text fields (F5). Finally, the results reported in Table 8 correspond to feature setting #4, where the set of features is composed by the 22 bio-entities (F1), their annotated content (F2, F3) and the EC Numbers (F4) listed in the training set.

For example, using the sample sentence given in Section Methodology, the set of features in each setting is represented by the following:

• Feature setting #1 (F1): *Enzyme, Fungus, Substratespecificity*
• Feature setting #2 (F1+F4): *Enzyme, Fungus, Substratespecificity, 11499*
• Feature setting #3 (F5): *substrate, specificity, three, ligninase, isozymes, white, rot, fungus, Trametes, versicolor, investigated*
• Feature setting #4 (F1+F2+F3+F4): *Enzyme, Fungus, Substratespecificity, 11499, ligninase, Trametes versicolor*

**Figure 3:**
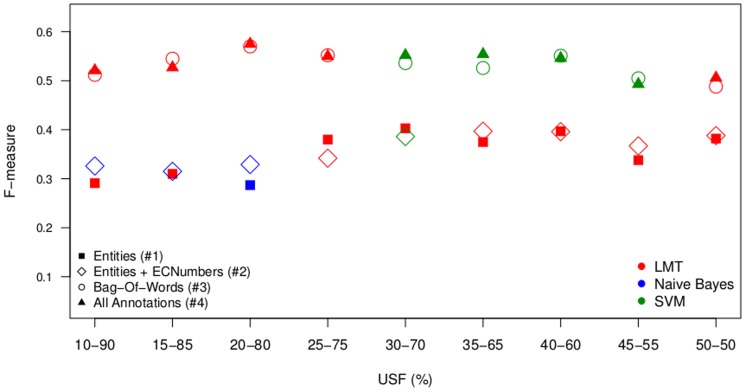
mycoSORT F-measure scores. Results of the Best Classifiers for Each Classification Model.

**Figure 4:**
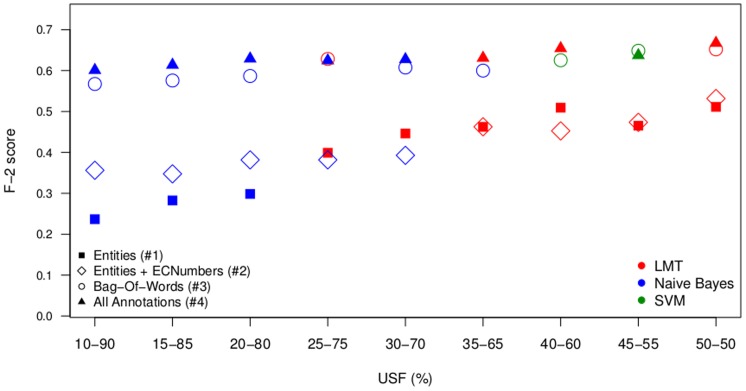
mycoSORT F-2 scores. Results of the Best Classifiers for Each Classification Model.

Overall 108 experiments were performed using the 3 learners, the 4 feature settings and the 9 under-sampling factors. Figs. 3 and 4 summarize the data of Tables 5 to 8 by showing the best feature settings with respect to the F-measure (Fig. 3) and F-2 score (Fig. 4).

## Discussion

The results presented in Table 7, with the use of features F5, are considered as the baseline for our experiments. These models use only a bag-of-words representation of the text as feature, without any domain annotation. When comparing the results in Table 7 (feature setting #3) with the results in Table 5 (feature setting #1) and Table 6 (feature setting #2), we observe that the scores of the bag-of-words approach are in general better than the scores using bio-entities, and bio-entities plus EC numbers as features. This difference can be explained by the feature space size of the models in Table 7 compared to the feature space size in models presented in Tables 5 and 6. While the classification models in Table 5 used only 22 features, the models in Table 6 used from 186 to 397 features across the different training sets, and the models in Table 7 used 7,622 to 20,729 features. Models with a larger feature space presented better performance, however the computational cost of the learning phase also increased greatly.

Analyzing the results presented in Tables 5 and 6, we can observe an improvement when the EC numbers are added to the set of features. Models presented in Table 8 show better performance for the majority of the under-sampling factors used. The feature space size of these models varies from 3,338 to 8,931; therefore they are smaller than the ones used for the models in Table 7, but still they are able to outperform the bag-of-words results. This indicates that the domain annotations do have a relevant discriminative power when classifying documents for the triage task.

It is also interesting to note that the use of only bio-entities as features also suggests an interesting cost-benefit. With a very concise feature space of only 22 bio-entities, the classification algorithm still manages to perform reasonably compared to the other more robust models. Such a compact feature space can be beneficial in circumstances in which computational cost and processing time are important concerns.

The results of different sampling factors used to generate the training corpora showed that the under-sampling of majority instances in the dataset contributed to enhance the classifier performance, confirming conclusions of previous works [25], [29]. Independently of the under-sampling factor used to create the training corpus, the test corpus was generated with a positive instance balance that corresponds to a real scenario experienced by biocurators.

The NB classifier was considered as a baseline algorithm in our experiments. However it outperformed LMT and SVM in models formed by highly-imbalanced datasets and a small feature space, such as 397 bio-entities and EC numbers. The SVM classifier outperformed in models that applied USF from 20% to 35%, and a larger feature space, with the use of domain annotations and bag-of-words. LMT outperformed the other classification algorithms in less imbalanced models, but with an even smaller feature space. When using an USF of 40% (which provides 50% of majority instances and 50% of minority instances), the LMT performance with 22 bio-entities as features was comparable to the performance of a model using 186 bio-entities and EC numbers. This confirmed the results previously described by [26] that LMT can handle harder classification tasks well.

## Conclusion

In this paper, we presented an evaluation of classification models to deal with document classification in the triage task. Usually, the triage task involves identifying very few relevant documents among a much larger universe of documents, hence datasets representative of this scenario have by nature an imbalance class distribution.

We evaluated different classification models in an attempt to identify the best configuration to be applied in a learning model to suitably tackle the triage task. Our intent was to develop a model that is capable of correctly classifying positive (relevant) instances, and at the same time reduces the misclassification of negative (not relevant) instances.

We experimented with 4 feature settings, 3 machine learning algorithms, and 9 under-sampling factors, for a total of 108 experiments. The system described in this paper can be applied to perform the literature triage of biomedical documents. The results demonstrate that, to achieve the best outcome, the most suitable approach for dealing with the triage of imbalanced corpora relies on a classification model composed by domain annotations, a balanced dataset and the use of LMT algorithm as classifier. Moreover, the other models studied here can be used as further options to tackle the document classification in the triage task, in case of existing constraints related to computational cost or data availability. The mycoSORT system is fully implemented, and publicly released as an open source toolkit available here: https://github.com/TsangLab/mycoSORT. The *mycoSet* corpus used in our experiment is also publicly available as a list of pairs [abstract PubMed ID - class of the abstract].

For further application of our techniques, we would like to point out that, besides mycoMINE, other scientific wide-ranging annotation schemas [33], [34] are available and could be used to support the triage task in different biomedical research contexts. Such alternatives use the Medical Subject Headings (MeSH) vocabulary, the Gene Ontology (GO) and the Unified Medical Language System (UMLS) thesaurus, being able to handle an extensive set of biomedical research subjects.

These tools can be helpful to provide broad-spectrum biomedical annotations for relevant units of text in a dataset. Later on, these annotations can play a similar role in the triage classification process as the mycoMINE annotations used as features in this work.

As future work, we plan to evaluate the presented classification models on the triage of medical related PubMed abstracts annotated with MeSH terms.
